# Delayed endometrial preparation for the induction of luteolysis as a potential factor for improved reproductive performance in Angus beef heifers with high antral follicle counts^†^

**DOI:** 10.1093/biolre/ioae146

**Published:** 2024-10-10

**Authors:** Martim Kaps, Lacey K Quail, Shelby L Rosasco, Alexandria P Snider, Saulo M Zoca, Kaitlin M Epperson, Jerica J J Rich, Jeremy R Miles, Matthew S Crouse, Brittney N Keel, Adam F Summers, George A Perry, Clay A Lents, Robert A Cushman

**Affiliations:** USDA, ARS, U.S. Meat Animal Research Center, Clay Center, NE, USA; Texas AgriLife, Overton, TX, USA; Department of Animal and Range Sciences, New Mexico State University, Las Cruces, NM, USA; USDA, ARS, U.S. Meat Animal Research Center, Clay Center, NE, USA; Department of Animal Science, South Dakota State University, Brookings, SD, USA; Texas AgriLife, Overton, TX, USA; Department of Animal Science, South Dakota State University, Brookings, SD, USA; USDA, ARS, U.S. Meat Animal Research Center, Clay Center, NE, USA; USDA, ARS, U.S. Meat Animal Research Center, Clay Center, NE, USA; USDA, ARS, U.S. Meat Animal Research Center, Clay Center, NE, USA; Department of Animal and Range Sciences, New Mexico State University, Las Cruces, NM, USA; Texas AgriLife, Overton, TX, USA; USDA, ARS, U.S. Meat Animal Research Center, Clay Center, NE, USA; USDA, ARS, U.S. Meat Animal Research Center, Clay Center, NE, USA

**Keywords:** antral follicle count, anti-Müllerian hormone, progesterone, conceptus elongation, oxytocin receptor, luteolysis

## Abstract

Antral follicle count (AFC) and anti-Müllerian hormone (AMH) concentrations are reflective for ovarian reserve and have been associated with improved reproductive performance in cattle. Key events for regulation of uterine receptivity are orchestrated by progesterone. As progesterone concentrations are greater in animals with high than low AFC, we tested the hypothesis, if the resulting improved uterine environment will lead to improved conceptus elongation and endometrial response to interferon tau. For four years, 10 heifers with lowest and highest AFC, respectively, were selected from 120 heifers. Reproductive tracts and blood samples for progesterone and AMH analysis were collected after synchronization and insemination. For a recovered conceptus, length was determined, and interferon tau (*IFNT*) transcript abundance was analyzed. Endometrial transcript abundance of interferon-stimulated gene 15 (*ISG15*) and oxytocin receptor (*OXTR*) were analyzed. Progesterone concentrations did not differ between low and high AFC groups (*P* = 0.1). A difference in conceptus length was not observed. Endometrial abundance of *ISG15* did not differ between pregnant low and high AFC heifers. Abundance of *OXTR* was greater in open low AFC than open high AFC heifers (*P* < 0.01). Interaction of AMH and pregnancy status was determined, with greater AMH in pregnant than open high AFC heifers (*P* < 0.05). Improved uterine environment in high vs. low AFC heifers did not result in longer conceptuses or improved endometrial response. As the increase in *OXTR* transcript abundance was only detected in low AFC heifers, reported differences in reproductive performance might be associated with earlier initiation of luteolysis.

## Teaser

Up-regulation of endometrial oxytocin receptor transcription is a prerequisite for the initiation of luteolysis and occurs later in heifers with increased ovarian reserve indicating temporal differences in the initiation of luteolysis.

## Graphical Abstract

**Graphical Abstract:**
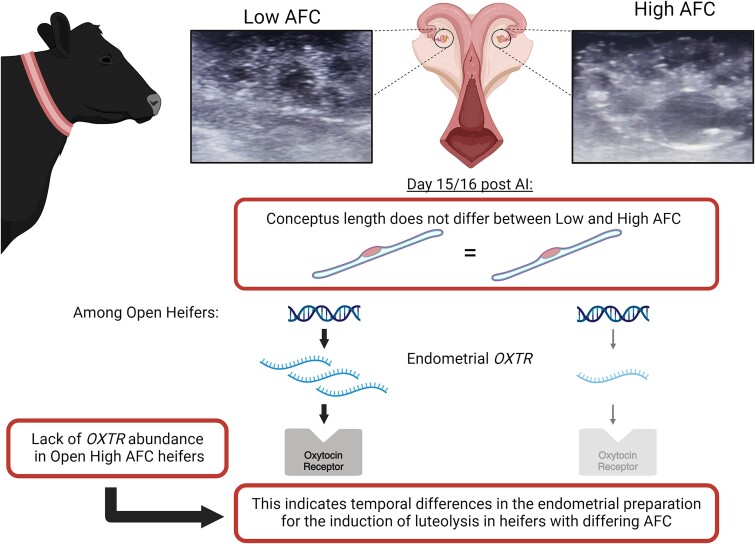

## Introduction

Selection of replacement heifers based on ultrasonographic determination of antral follicle count (AFC) has been suggested as a potential strategy to increase reproductive efficiency of cow-calf operations [1–3]. Ultrasonographic assessment of AFC can be easily performed by a trained technician at any stage of the estrous cycle [2] is repeatable within individuals [4–6] and predictive of the size of the ovarian reserve [7]. Heifers with a greater number of antral follicles conceived earlier during their first breeding season compared to their contemporary herd mates with fewer follicles [3, 8, 9]. Consequently, heifers with greater numbers of antral follicles give birth earlier, produce calves with greater weaning weights and remain productive within the herd for a greater number of years [8, 10]. The overall economic benefit of selecting replacement heifers with greater numbers of antral follicles is, therefore, mainly attributed to increased lifetime productivity [10–12].

A major contributor to reduced reproductive efficiency in cattle is the high percentage of early embryonic losses prior to, or around maternal recognition of pregnancy [13, 14] with failure of conceptus elongation as a key factor related to early pregnancy loss. During the early and mid-luteal phase, temporal changes in endometrial gene expression are orchestrated by progesterone and ensure a uterine environment suitable for the support of embryonic development and elongation [15]. Increased concentrations of progesterone, either naturally or achieved by exogenous supplementation, enhance conceptus elongation [16], whereas reduced concentrations of progesterone hamper embryonic development [17]. Those effects on conceptus elongation are evoked indirectly by the action of progesterone on the uterine endometrium [18] and changes in composition of the histotroph secreted by the uterine glands [17, 19]. Cattle with increased numbers of antral follicles have greater luteal phase concentrations of progesterone compared to cattle with diminished numbers of antral follicles [2, 20, 21]. The question arises, if greater circulating concentrations of progesterone in heifers with high AFC result in a more supportive uterine environment for embryo elongation, leading to improvements in conceptus development as estimated by conceptus length. These differences could contribute to improved embryonic survival and reduce the incidence of embryonic loss. Overall, that might explain the improved reproductive performance of cattle with high numbers of antral follicles via an optimized process of maternal recognition and establishment of pregnancy. This concept is supported by previous investigations on composition of uterine histotroph in heifers differing in AFC. Heifers with increased number of antral follicles had greater concentrations of glucose and protein in uterine luminal fluid on day 16 after estrus than their low AFC counterparts [5, 22]. Along with lipids and nucleotides, glucose and proteins are the main metabolites driving conceptus elongation and development.

With increasing conceptus length, the magnitude of embryonic interferon tau (IFNT) signaling increases [23, 24]. In consequence to a stronger embryo-derived signal, a more pronounced endometrial response to the presence of an embryo can thus be expected, when embryonic signaling is increased proportional to conceptus length. A more pronounced increase in interferon-stimulated genes could, therefore, be expected and further contribute to a more effective inhibition of events preceding initiation of luteolysis, such as up-regulation of oxytocin receptors in the endometrial epithelium.

The aim of this study was, therefore, to investigate the potential for differences in conceptus length resulting from described differences in progesterone concentration and uterine glucose concentration between beef heifers with differing numbers of antral follicles. Furthermore, we assessed, if transcription patterns of genes involved in embryo–maternal signaling differed accordingly. We hypothesized that conceptuses found in heifers with more antral follicles will be longer than in heifers with diminished number of antral follicles. Under the assumption that IFNT expression is proportional to conceptus length, we further hypothesized that presence of a longer conceptus will be reflected in an increased transcript abundance of interferon-stimulated gene 15 (*ISG15*) in the endometrium.

## Material and methods

### Heifer management

All animal procedures were approved by the U.S. Meat Animal Research Center (USMARC) Institutional Animal Care and Use Committee (Experimental Outline # 94.1). Angus heifers (*n* = 120/year for 4 years, 2018, 2019, 2021, and 2022) were weaned to the USMARC feedlot and provided ad libitum access to a standard heifer development diet for a target of 55% of mature body weight by breeding. In the following March, at the age of approximately 11 months (334.8 ± 17.9 S.D. days), the number of antral follicles and the presence of corpora lutea (CL) was determined by transrectal ultrasonographic exam using an Aloka 500 SD ultrasound machine equipped with a 7.5 Mhz linear probe (Aloka, Wallingford, CT, USA). At this time heifers were fitted with accelerometers (HeatTime®Pro, Allflex Global, Kenilworth, NJ, USA) for continuous monitoring of behavioral estrus. A second ultrasonographic examination, with collection of the identical information, was conducted again 1 month later (364.3 ± 17.8 S.D. days of age). Each year, after the second ultrasonographic exam, 10 cyclic animals with the greatest (high AFC, 10/year, total *n* = 40) and lowest AFC (low AFC, 10/year, total *n* = 40) were selected to be synchronized by a 7-day CO-Synch protocol and artificially inseminated 48 h after administration of a prostaglandin F_2α_ analog (25 mg, Lutalyse®, Zoetis, Florham Park, NJ, USA) with a standard artificial insemination (AI) dose of a single bull of proven fertility. On day 15 or 16 after AI, selected heifers were sent to the USMARC abattoir, blood samples were collected at exsanguination and reproductive tracts were collected and returned to the laboratory within 15 min. Heifers harvested on day 15 and 16 after AI were equally distributed between high and low AFC groups.

### Reproductive tract measurements, conceptus recovery, and sample collection

Upon arrival of the reproductive tract at the laboratory, the broad ligament and any extra adipose tissues were trimmed, and the vagina was trimmed even with the external cervical os for consistency of uterine weights between heifers. Uterine weight was determined, and ovaries were removed, weighed and assessed as previously described [25]. Briefly, the number of small, medium, and large follicles (<5, 5–10, and >10 mm) on each ovary was counted and the number and location of all CL were recorded. The diameter of the largest follicle on each ovary was measured using a Vernier caliper, and the CL was dissected and weighed. A cross-section of the ovary that did not contain the active CL was fixed in at least the 10-fold volume of neutral buffered formalin (10%) for subsequent histological evaluation of pre-antral follicle numbers.

For conceptus recovery, the uterine lumen was flushed as previously described [26] with slight modifications. Briefly, the uterine lumen was clamped at the cervix and 20 ml phosphate buffered saline was injected into a small incision at the uterotubal junction of the uterine horn contralateral to the location of the CL. The incision was clamped to prevent fluid loss. The uterus was massaged for equal fluid distribution and the fluid was recovered from the opposite uterine horn into a 100-mm petri dish. The recovered fluid was searched for the presence of a conceptus using a stereomicroscope (Nikon; Melville, NY) at 10x magnification. A detected conceptus was manually detangled, and length was measured using a transparent scale bar. The embryonic disc was manually dissected with a razor blade from the trophectoderm. Both specimens of the conceptus were snap frozen individually and stored at −80°C until further analysis. For a subset of heifers (*n* = 40, first two years of the study) glucose concentration in uterine luminal fluid was measured, these data have previously been published [22]. Uterine horns were dissected in a transversal plane 1 cm anterior to the bifurcation and endometrium diameter was measured using a Vernier caliper [5]. After further longitudinal opening of the horn, caruncular and intercaruncular endometrium were excised from the horn ipsilateral to the location of the CL, snap-frozen and stored at −80°C until further analysis.

### RNA extraction

Total cellular RNA was extracted from 15 mg of endometrial tissue and total collected trophectoderm tissue using the Qiagen RNAeasy mini kit (Qiagen Inc., Valencia, CA, USA) with on column DNAse treatment following the manufacturer’s protocol. Quantity of RNA was assessed by Nanodrop 8000 spectrophotometer (Thermo Fisher Scientific, Wilmington, DE, USA). The RNA integrity was assessed using the Agilent 2200 screentape assay (Applied Biosystems, Carlsbad, CA, USA). Mean RNA integrity number was 8.5 ± 0.5 S.D. for all endometrial and 9.6 ± 0.3 S.D. for all trophectoderm samples. Following extractions, a total of 1 μg RNA per sample was reverse transcribed to cDNA using the iScript Reverse Transcription kit (Bio-Rad Laboratories, Hercules, CA, USA) and cDNA was diluted to a final working concentration of 2.5 ng/μl.

### Quantitative real-time PCR

Quantitative real-time PCR (qPCR) assays were performed in duplicates, using previously validated primer sets for *IFNT*, *ISG15*, *OXTR,* and *GAPDH*, described in Table 1. Two microliters of RNA-equivalent cDNA (5 ng total) were added to a 20-μl reaction containing 10 μl of iTaq Universal SYBR Green Supermix (BioRad Laboratories, Hercules, CA, USA), 6 μl of nuclease-free water and 1 μl each of the appropriate forward and reverse primers (10 μM). Analysis was performed on a CFX96 Realtime System (BioRad Laboratories, Hercules, CA, USA).

**Table 1:** Genes, primer sequences, and annealing temperatures for genes amplified during RT-PCR in endometrium (*OXTR, ISG15*) and trophectoderm (*IFNT*).

Gene	Primer	Sequence	Annealing temperature	Reference	Gene accession number
*OXTR*	Forward	5′-CGTGCAGATGTGGAGTGTCT-3′	55°C	Perry et al. (2020) [27]	NM_174134.2
	Reverse	5′-CCTATCAGTCACAGCGTGGA-3′			
*ISG15*	Forward	5′-GGTATCCGAGCTGAAGCAGTT-3′	62°C	Han et al. (2006) [28]	NM_174366.1
	Reverse	5′-ACCTCCCTGCTGTCAAGGT-3′			
*IFNT*	Forward	5′-GCTATCTCTGTGCTCCATGAGATG-3′	58°C	Shorten et al. (2018) [29]	NM_001015511.4
	Reverse	5′-AGTGAGTTCAGATCTCCACCCATC-3′			
*GAPDH*	Forward	5′- GATTGTCAGCAATGCCTCCT-3′	61°C	Han et al. (2006) [28]	NM_001034034. 2
	Reverse	5′-GGTCATAAGTCCCTCCACGA-3′			

Conditions for PCR included 5 min of denaturation at 95°C followed by amplification 95°C for 15 s, annealing for 15 s, and extension at 70°C for 15 s for 40 cycles using the annealing temperatures provided in Table 1. A single melting curve peak was confirmed for all primer pairs. Raw Cq values for GAPDH were stable in the statistical model (see below), confirming the validity of GAPDH as an endogenous reference gene for this study. Nuclease-free water was used as a negative control to exclude background contamination. Intra-assay coefficients of variation for GAPDH and the target genes were ≤ 20%. Relative transcript abundance was determined following the 2^-ΔΔCT^ method [30]. The sample with the lowest normalized amount of expression was used as the calibrator (set to 1) and remaining samples are displayed as fold changes to the calibrator [31].

### Analysis of reproductive hormones

Blood samples were collected at exsanguination into clot-activator containing tubes. After clotting, tubes were centrifuged at 1000 × *g* for 30 min and serum was stored at −20°C until further analysis. For analysis of progesterone concentrations, a previously described and validated radioimmunoassay [32] was used. Intra- and inter-assay CVs for progesterone were 4.0% and 4.4%, respectively, and assay sensitivity was 0.4 pg/ml. Serum concentrations of anti-Müllerian hormone (AMH) were analyzed using a commercial bovine AMH enzyme-linked immunosorbent assay (Ansh Labs, Webster, TX, USA) according to manufacturers’ instructions. The sensitivity of the AMH assay was 11 pg/mL and the mean intra- and inter-assay coefficients of variation were 3.0% and 3.8%, respectively.

### Ovarian histology

Formalin-fixed ovarian cross sections were embedded in paraffin and sectioned into 5 μm thick slices. Five sections, at least 50 μm apart, were sectioned and stained using hematoxylin and eosin. The number of primordial (oocyte surrounded by a single layer of squamous pregranulosa cells), primary (oocyte surrounded by a single layer of cubic granulosa cells) and secondary (oocyte surrounded by multiple layers of granulosa cells) follicles in the ovarian cortex was determined at 200x magnification, according to the previously established criteria [33, 34].

### Statistical analysis

Data for different methods of follicle count determination (sonographic, surface count, microscopic counts), reproductive tract weight, and ovary size and weight were analyzed using the MIXED procedure for ANOVA in SAS (SAS Inst. Inc., Cary, NC, USA) with AFC group as a fixed effect. Data for hormonal analysis (progesterone and AMH) and abundance of *ISG15* and *OXTR* transcript were also analyzed by the MIXED procedure for ANOVA. The model included the fixed effects of AFC group (low or high), pregnancy status (open or pregnant) and their interaction with the linear effect of day (15 or 16) included as a co-variate. Conceptus length and *IFNT* transcript abundance were analyzed using the MIXED procedure for ANOVA with day, AFC group, and the interaction as fixed effects. Where applicable, pairwise comparison (PDIFF) was applied for post hoc analysis. Pregnancy rate, response to synchronization, and incidence of double ovulation were analyzed by Fishers exact test. Correlations were analyzed using the CORR procedure in SAS. Data are presented as least squares mean ± S.E.M. A *P*-value ≤0.05 was considered significant, *P*-values between 0.05 and 0.10 were considered a tendency. Animals with double ovulations (*n* = 4) and heifers that failed to ovulate (*n* = 9), were excluded from statistical analysis of progesterone concentration and analyses involving pregnancy status. Pregnancy was defined by presence of a conceptus recovered by uterine flushing. In one animal, no conceptus was detected in the uterine flush, whereas relative *ISG15* transcript abundance was increased more than 300-fold. It cannot be completely excluded, that a conceptus was present but not detected. As determination of pregnancy status for this animal was not possible, it was excluded from all analyses including pregnancy status. A second animal had a conceptus-like structure that best resembled a degenerated, non-viable conceptus from which no RNA could be extracted, and that additionally lacked increased endometrial expression of *ISG15.* This heifer and was defined as nonpregnant.

## Results

**Figure 1:**
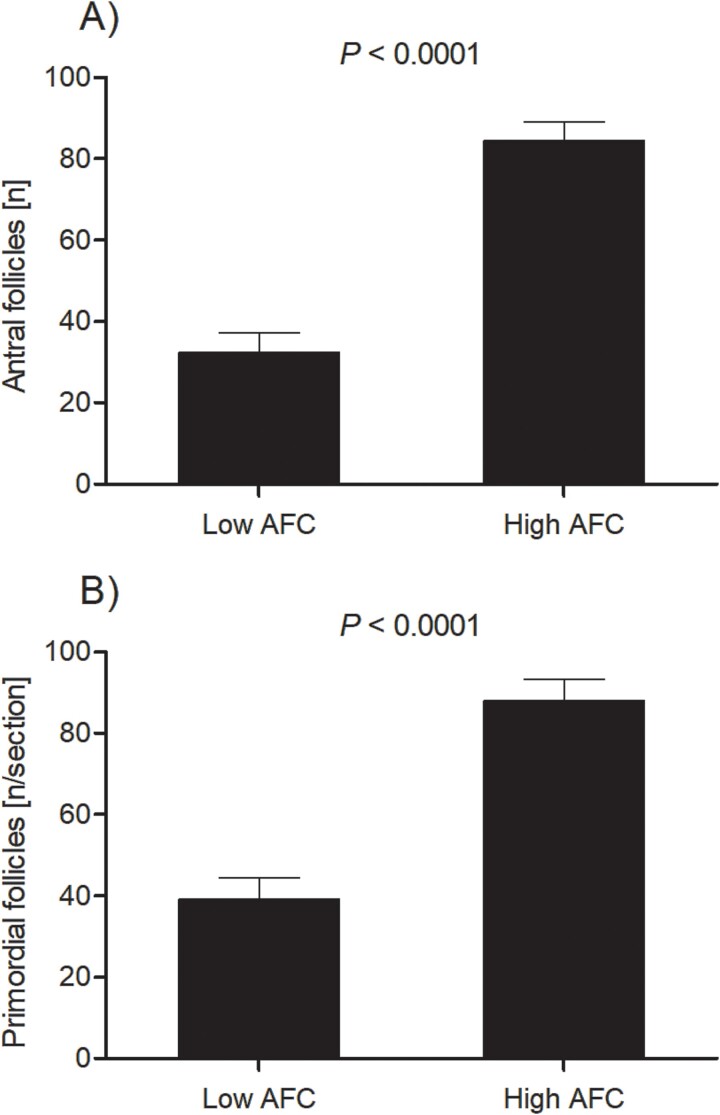
Mean number of primordial follicles per histology section (A) and mean number of follicles on the ovarian surface (B) for the high and low AFC groups. Data are presented as LS mean ± SEM. The *P*-value for the main effect of AFC group is provided in the figure.

The average number of antral follicles detectable by ultrasonography within a heifer across the two examinations differed between AFC groups (*P* < 0.0001). In low AFC heifers, 13.7 ± 0.7 follicles were detectable, while in high AFC heifers, 35.2 ± 0.7 follicles were detectable across the two examinations. In accordance with ultrasonography, the number of follicles present on the ovarian surface after collection of the reproductive tracts differed between AFC groups (*P* < 0.0001, Figure 1A). Furthermore, there was a greater number of primordial follicles per histological section in heifers from the high AFC group compared to heifers from the low AFC group (*P* < 0.0001; Figure 1B). The number of primary and secondary follicles per histological section differed due to AFC group, with 6.2 ± 1.8 primary follicles per section in ovaries of low AFC compared to 13.4 ± 1.8 in ovaries from high AFC heifers (*P* < 0.01). The number of secondary follicles was 1.0 ± 0.2 in low AFC compared to 1.8 ± 0.2 in high AFC group (*P* < 0.01). Ovaries from the high AFC group were larger and weighed more than ovaries from heifers in the low AFC group (*P* < 0.001; Table 2); however, weight of the reproductive tract did not differ between AFC groups (*P* > 0.80).

**Table 2:** Ovarian response to synchronization protocol and pregnancy rate.

	Low AFC	High AFC	*P*-value
Ovary Length (mm)^1^ Height (mm)^1^ Weight (g)^1^ Reproductive tract weight (g)	25.5 ± 0.5 15.9 ± 0.5 3.7 ± 0.2 232.1 ± 7.4	29.6 ± 0.5 19.2 ± 0.5 5.2 ± 0.2 230.4 ± 7.4	<0.0001 <0.0001 <0.0001 0.87
Ovulation	35/40 (87.5%)	36/40 (90%)	1.00
Double ovulation	3/35 (8.6%)	1/36 (2.8%)	0.36
Pregnant (Among heifers that responded to synchronization)	16/34 (47.1%)	16/36 (44.4%)	1.00

^1^Mean of left and right ovary

**Figure 2:**
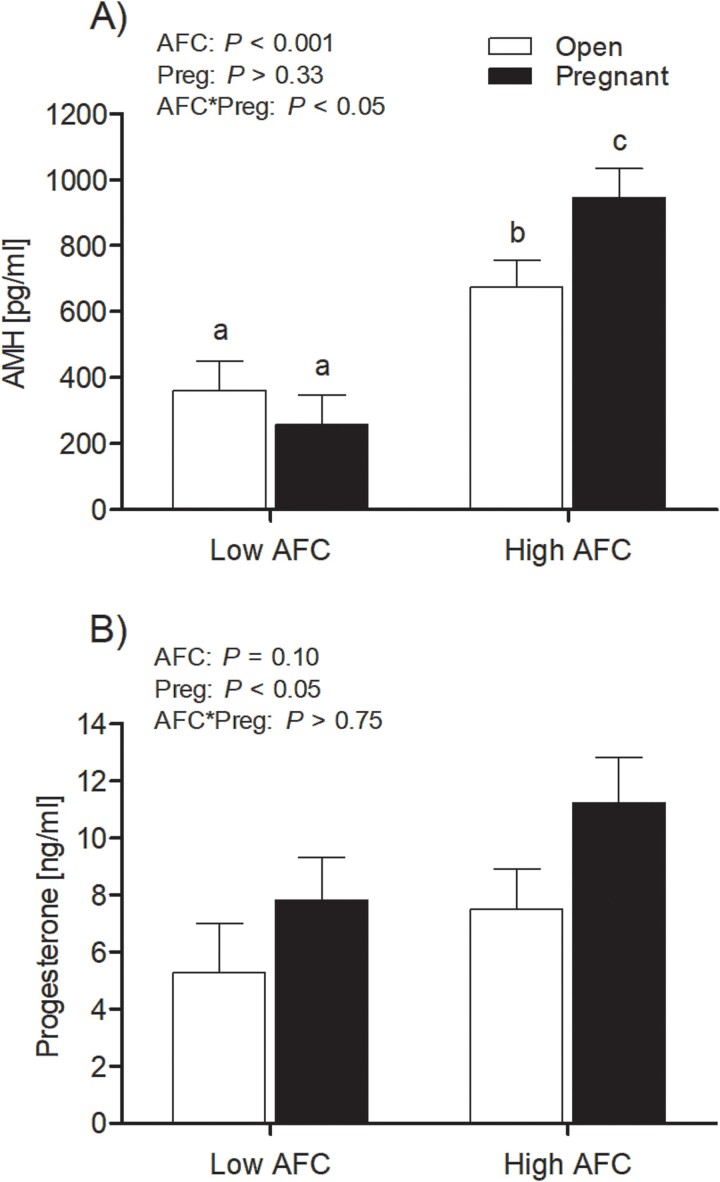
Least square means for AMH serum concentration (A) and progesterone serum concentration (B) for open and pregnant heifers in high and low AFC groups, respectively. The *P*-values for the main effect of group, pregnancy status, and their interaction are provided in the figure.

There was a significant interaction of AFC group and pregnancy status for circulating concentrations of AMH (*P* < 0.05; Figure 2A). Concentrations of AMH were least in heifers from the low AFC group and did not differ due to pregnancy status, whereas concentrations of AMH were intermediate for open heifers in the high AFC group and greatest for pregnant heifers in the high AFC group (Figure 2A). Strong to moderate correlations were observed for plasma concentrations of AMH, average ultrasonographic AFC, surface count and the number of primordial follicles/section (*P* < 0.0001; Table 3). The interaction of AFC group and pregnancy status did not influence circulating concentrations of progesterone (*P* > 0.70; Figure 2B); however, circulating concentrations of progesterone were greater in pregnant heifers than open heifers (*P* < 0.05) and numerically greater in heifers from the high AFC group than heifers from the low AFC group (*P* = 0.10).

**Table 3:** Pearson correlation coefficients between AMH and different stages of follicular development (level of significance is given in parenthesis).

	Sonographic AFC^1^	Surface count	Primordial	Microscopy primary	Secondary
AMH	0.66 (<0.0001)	0.81 (<0.0001)	0.47 (<0.0001)	0.15 (0.18)	0.09 (0.43)
Sonographic AFC		0.71 (<0.0001)	0.60 (<0.0001)	0.44 (<0.0001)	0.39 (<0.001)
Surface count			0.55 (<0.0001)	0.38 (<0.001)	0.30 (<0.01)
Primordial				0.55 (<0.0001)	0.50 (<0.0001)
Primary					0.61 (<0.0001)

^1^Mean AFC of first and second sonography

**Figure 3:**
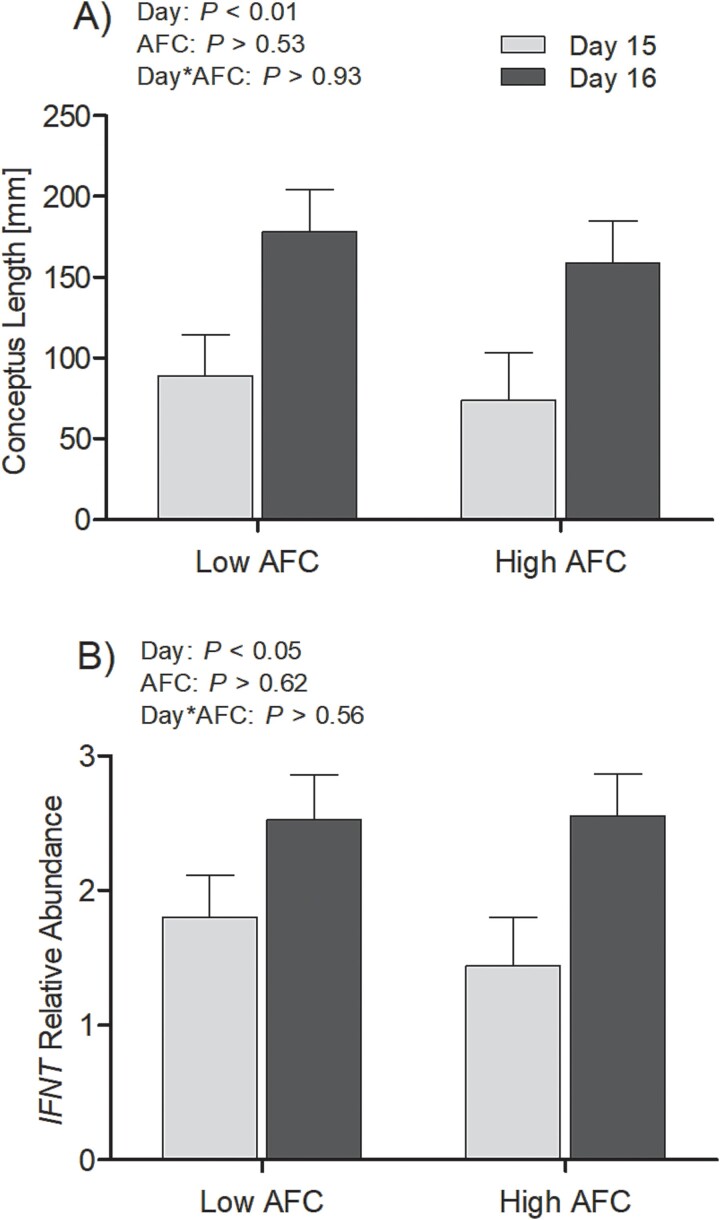
Least square means for (A) conceptus length on days 15 and 16 after AI for groups high and low AFC and (B) the corresponding relative increase in trophectodermal *IFNT* transcript abundance. The *P*-values for the main effect of group, pregnancy status, and their interaction are provided in the figure.

Pregnancy rate, ovulatory response to the synchronization protocol and the incidence of double ovulations did not differ between AFC groups (*P* > 0.36, Table 2). Among heifers that conceived, there was no significant interaction of AFC group and day for conceptus length or transcript abundance of *IFNT* transcript in the trophectoderm (*P* > 0.55); however, both variables were affected by Day (*P* < 0.01), such that conceptus length and *IFNT* transcript abundance were greater on Day 16 compared to Day 15 (Figure 3A and B). No difference in conceptus length and *IFNT* transcript abundance was observed between AFC groups (*P* > 0.5). A moderate correlation of conceptus length and *IFNT* transcript abundance (*R* = 0.51, *P* = 0.01) was observed.

**Figure 4:**
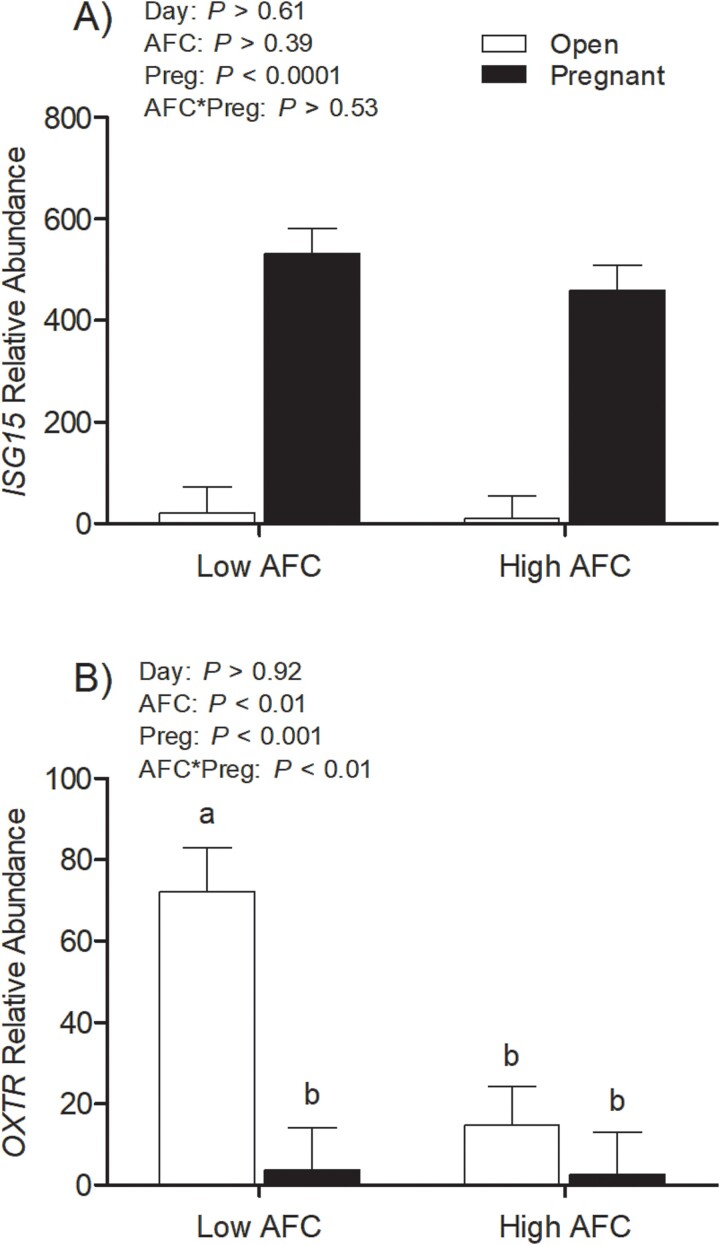
Least square means of relative *ISG15* (A) and *OXTR* (B) transcript abundance in endometrium for open and pregnant heifers within high and low AFC groups. The *P*-values for the main effect of group, pregnancy status, and their interaction are provided in the figure.

The interaction of AFC group and pregnancy status was not significant for relative transcript abundance of *ISG15* in the endometrium (*P* > 0.50, Figure 4A). Pregnant heifers had greater *ISG15* transcript abundance than Open heifers (*P* < 0.001) but *ISG15* transcript abundance did not differ between the high and low AFC groups (*P* > 0.35). There was a moderate to weak correlation of endometrial *ISG15* transcript abundance with conceptus length (*R* = 0.48, *P* < 0.01) and *IFNT* transcript abundance in the trophectoderm (*R* = 0.37, *P* < 0.05), respectively. For *OXTR*, an interaction of AFC group and pregnancy status was observed (*P* < 0.001), with increased *OXTR* transcript abundance only present in open heifers from the low AFC group but not in open heifers from the high AFC group, or pregnant heifers from either AFC group (*P* < 0.001, Figure 4B). There was a moderate negative correlation of *OXTR* transcript abundance to serum concentration of progesterone among low AFC heifers (*R* = 0.53, *P* = 0.01), but not for high AFC heifers (*P* > 0.15).

## Discussion

The results of the present study do not support the hypothesis that a more supportive nutrient environment in the uterus would result in longer conceptuses in heifers with greater numbers of antral follicles. The hypothesized differences in maternal response to embryonic signaling (*ISG15*), due to differences in conceptus length were also not observed. However, a greater endometrial *OXTR* transcript abundance was observed in Open heifers with diminished numbers of antral follicles compared to heifers with increased numbers. These results thus imply that the prerequisites for the initiation of luteolysis are given later in heifers with greater ovarian reserve.

The difference in the transcript abundance for *OXTR* between open heifers with increased AFC compared to open low AFC heifers is an interesting result that was unexpected. An increase in *OXTR* transcript abundance in the endometrium is among the earliest indicators of the initiation of luteolysis in ruminants [35, 36]. Our results thus indicate that initiation of luteolysis might be delayed in heifers with enhanced ovarian reserve, potentially resulting in a prolonged window for maternal recognition. In pregnant cows and sheep, OXTR expression is inhibited by the presence of embryo derived IFNT [37], but the understanding of mechanisms regulating the timing of OXTR upregulation remains incomplete. It is known, that an increase in *OXTR* transcript abundance and luteolysis occur earlier in two-wave than three-wave estrous cycles in cattle, resulting in differences estrous cycle length [36]. The proportion of cattle with two- and three- wave estrous cycles did not differ between groups with high and low AFC in heifers in a previous study [4]. In women a relation of AFC and length of the menstrual cycle has been described, with shorter cycles in women with diminished AFC [38]. In contrast, we recently demonstrated that among heifers the mean AFC does not change with increasing or decreasing length of the estrous cycle [39]. It seems, therefore, unlikely that there was an overrepresentation of two-wave heifers with shorter estrous cycles in the low AFC group. A steeper slope in the decrease of circulating progesterone concentrations has been described in cows with greater numbers of follicles [20]. In consequence, the delayed initiation of luteolysis in the high AFC heifers that is suggested by the presented findings, must not necessarily be accompanied with an increase in estrous cycle length, as luteolysis might occur more rapidly at a later timepoint of the estrous cycle.

The necessity of estradiol for the upregulation of OXTR in the endometrial luminal epithelium has previously been questioned [35, 40]. However, a promoting effect of estradiol on spontaneous OXTR upregulation has been described in vitro [40]. In addition, more recent studies clearly indicate, that upregulation of oxytocin receptor can be induced by administering estradiol on day 15 of the estrous cycle in cattle [41, 42]. In both studies, luteolysis was advanced by estradiol treatment on day 15 of the estrous cycle. In follicles dissected at an early stage of the follicular wave greater concentration of estradiol in follicular fluid and greater abundance of *CYP19A1* in granulosa cells have been described in low AFC than high AFC heifers [43]. It is thus likely, that differences in the steroidogenic capacity of follicles within a non-ovulatory follicular wave exist between heifers of differing AFC. The resulting increase of endogenous estradiol could consequently advance OXTR upregulation and the initiation of luteolysis in low AFC heifers. Future studies investigating the effect of estradiol derived by growing follicles of a follicular wave around the window of maternal recognition of pregnancy in heifers of differing AFC might thus enable us to further characterize the role of estradiol in the temporal dynamics of OXTR expression and luteolysis in cattle. In addition to differing estradiol concentrations, the duration and intensity of the inhibitory effect of progesterone on OXTR expression [44] might also contribute to differences in OXTR expression between low and high AFC heifers.

Circulating progesterone concentrations are greater in heifers with greater AFC [2, 20, 21]. In the present study, the difference in progesterone concentrations between heifers with high and low AFC did not reach statistical significance. In a previous study, progesterone concentrations were greater between day three and 14 of the estrous cycle [20]. The timing of assessment of serum progesterone concentrations was thus most likely too late in the estrous cycle to detect significant differences between AFC groups. Despite that, glucose concentration in uterine luminal fluid was assessed in a subset of heifers included into this study, and was greater in high AFC than low AFC heifers [22]. Progesterone is the major regulator of uterine receptivity and composition of uterine histiotroph [17, 19]. In the context with previous reports of greater progesterone concentrations in high AFC heifers, it is thus highly likely that differences in progesterone were present during earlier stages of the estrous cycle in heifers of the current study.

The exact reasons for increased progesterone concentrations in heifers with increased AFC have not yet been fully determined, but a compromised ability of granulosa cells in the dominant follicle of heifers with low AFC to undergo luteinization, as well as chronically high LH and FSH concentrations interfering with granulosa cell differentiation, have been proposed as potential reasons [20]. Nevertheless, it is common consensus that increased progesterone concentrations positively affect conceptus elongation mediated exclusively via the endometrium [16, 18, 45, 46]. It was thus unexpected, that conceptus length did not differ between AFC groups. In most of the studies investigating the relation of conceptus length and progesterone, exogenous administration of progesterone early during the luteal phase was part of the experimental design. Endometrial changes in response to increased progesterone might differ depending on the source and dose of progesterone. In heifers with greater ovarian reserve, the potentially prolonged period before initiation of luteolysis might be more important for establishment of pregnancy than conceptus length itself. In certain cases where a conceptus is short for conceptional age, this prolonged period might be sufficient to achieve a greater and more adequate expression of IFNT by the conceptus to suppress luteolytic mechanisms and maintain pregnancy.

As in previous studies [47, 48], we observed greater progesterone concentrations in pregnant than in open heifers. This can be attributed to different reasons. First, among open heifers the initiation of luteolysis could already have occurred and could be causative for reduced progesterone concentrations. With greater endometrial *OXTR* abundance in open low AFC heifers than any other subgroup, this mechanism might be more relevant in low AFC heifers. This finding is further supported by the negative correlation of *OXTR* abundance and plasma progesterone found in low AFC, but not in high AFC animals. Second, conceptus derived IFNT has been reported to exert direct effects on the CL, such as increasing expression of interferon-stimulated genes in sheep [49]. Furthermore, transcriptomic analysis in bovine CL tissue recently revealed up-regulation of pathways expected to enhance or sustain CL function around the window of maternal recognition [50], which potentially contribute to greater progesterone concentrations in pregnant heifers.

In accordance with previous studies [23, 24, 51], we observed a strong increase in conceptus length between days 15 and 16 of pregnancy and an increase of *IFNT* transcript abundance from days 15 to 16. Conceptus length and *IFNT* transcript abundance were moderately correlated to endometrial *ISG15* transcript abundance. An interesting finding is, therefore, that conceptus length and *IFNT* transcript abundance were greater on day 16 compared to day 15, but *ISG15* transcript abundance did not increase similarly between days 15 and 16 in the endometrium of pregnant heifers. This is in contrast, to recently described increased endometrial response of classical interferon-stimulated genes (ISG15, OAS1, MX1, and MX2) to increasing doses of IFNT in endometrial explants from late estrus [52]. The results of our study, thus, imply the presence of a maximum level of *ISG15* transcript abundance in the endometrium in response to embryo derived IFNT in vivo. It appears that, even if embryonic *IFNT* transcript abundance further increases between days 15 and 16, the endometrial response regarding *ISG15* transcript abundance does not further increase at the transcriptional level. An alternative explanation is a potential time lag between the increase in *IFNT* abundance and the stimulatory effect of IFNT on *ISG-15* transcription.

Plasma concentrations of AMH in the current study were within the previously described ranges [53, 54] and none of the heifers were below the detection limit of the assay. As expected, a clear association of antral follicle count and ovarian reserve with mean plasma concentration of AMH was observed. As in previous reports [2, 53, 55], the strong relation between AMH and ovarian reserve was confirmed. A novel finding in this study is the interaction of AFC and pregnancy status, which adds new information to the relation of AMH, AFC, and fertility. Over the last decade, this relation has been reported to be more complex than initially anticipated [7]. Controversial results for the relation of AMH, AFC, and fertility have been described in cattle (reviewed by [56]) and sheep [57]. Also, greater AMH concentrations are not necessarily accompanied with greater fertility, and a quadratic instead of linear relation between AMH and fertility has thus been suggested and reported [58, 59]. Our results indicate that among heifers with low AFC, additional testing for AMH concentration does not result in further information about fertility potential compared to ultrasonographic determination of AFC alone. In contrast, among heifers with high AFC further testing for AMH might result in additional information regarding fertility potential with greater probability of pregnancy for heifers with greater AMH concentrations. The greater variability in AMH concentration among high AFC heifers might be the prerequisite to allow for differentiation of heifers with differing intrinsic potential to become pregnant. In addition to factors that improve establishment of pregnancy via the alteration of uterine environment, a potential mechanism for increased establishment of pregnancy among heifers with greater AFC or AMH might be a direct link between AMH and developmental competence of the oocytes.

For the present study, heifers that did not ovulate in response to the CO-Synch protocol were excluded from the analysis. It has recently been reported that heifers with lower AMH concentrations respond better to synchronization protocols than their high AMH counterparts [60]. Furthermore, improved response to synchronization with regard to estrous behavior has been determined for low AFC heifers [54]. While in the same study, pregnancy rate was greater in cows with high AMH compared to low AMH heifers when natural service (no synchronization) was performed. Ribeiro et al. [54], therefore, suggested that synchronization might override the positive effects of AMH on fertility. The potentially greater fertility in High AFC heifers with greater AMH concentrations, might thus be overseen in datasets, where ovulation rate in response to synchronization is unknown or not considered. Exclusion of non-ovulating animals might, therefore, be a strategy to correct for the disturbance factor of ovulatory response and a potential explanation for yet inconclusive reports regarding heifer fertility and AMH.

In conclusion, improved endometrial glucose concentration in heifers with greater AFC did not result in greater conceptus length or an intensified embryo-maternal crosstalk. The observed differences in endometrial upregulation of *OXTR* indicate temporal differences in the preparation of the endometrium for induction of luteolysis. This might result in a delayed of the initiation of luteolyis and a wider window for maternal recognition in High AFC animals that could contribute to improved reproductive performance. The exact mechanism regulating the differential timing of *OXTR* upregulation between heifers differing in AFC remains to be investigated. This knowledge might contribute to further characterize the so far controversial relation between ovarian reserve and fertility potential in cattle. Our results further suggest that the combined determination of AFC and AMH, at least for research purpose, would allow us to obtain a more detailed resolution of the actual relation of AMH, ovarian reserve and fertility especially among heifers with higher AFC.

## Data Availability

The data underlying this article will be shared on reasonable request to the corresponding author.
